# The Prognostic Value of the Novel Global Immune-Nutrition-Inflammation Index (GINI) in Stage IIIC Non-Small Cell Lung Cancer Patients Treated with Concurrent Chemoradiotherapy

**DOI:** 10.3390/cancers15184512

**Published:** 2023-09-11

**Authors:** Erkan Topkan, Ugur Selek, Berrin Pehlivan, Ahmet Kucuk, Duriye Ozturk, Beyza Sirin Ozdemir, Ali Ayberk Besen, Huseyin Mertsoylu

**Affiliations:** 1 Department of Radiation Oncology, Baskent University Medical Faculty, Adana 01120, Turkey; 2 Department of Radiation Oncology, Koc University School of Medicine, Istanbul 34010, Turkey; ugurselek@yahoo.com; 3 Department of Radiation Oncology, Bahcesehir University, Istanbul 34349, Turkey; berrinpelivan@gmail.com; 4 Clinic of Radiation Oncology, Mersin Education and Research Hospital, Mersin 33160, Turkey; drakucuk@yahoo.com; 5 Department of Radiation Oncology, Faculty of Medicine, Afyonkarahisar Health Sciences University, Afyonkarahisar 03030, Turkey; duriyeozturk07@gmail.com; 6 Clinic of Radiation Oncology, Medical Park Hospital, Antalya 07160, Turkey; beyza_sirin@hotmail.com; 7 Department of Medical Oncology, Medical Park Hospital, Adana 07160, Turkey; besenay@gmail.com; 8 Department of Medical Oncology, Istinye University, Istanbul 34010, Turkey; huseyinmertsoylu@gmail.com

**Keywords:** non-small cell lung cancer, biological marker, Global Immune-Nutrition-Inflammation Index, chemoradiotherapy, prognosis, survival

## Abstract

**Simple Summary:**

We investigated the prognostic significance of the newly created Global Immune-Nutrition-Inflammation Index (GINI) in IIIC non-small cell lung cancer (NSCLC) patients who received definitive concurrent chemoradiotherapy (CCRT). A total of 802 newly diagnosed stage IIIC NSCLC patients were included. The optimal pre-CCRT GINI cutoff was 1562 (area under the curve: 76.1%; sensitivity: 72.4%; specificity: 68.2%; Youden index: 0.406). GINI ≥ 1562 was associated with significantly shorter median locoregional progression-free (*p* < 0.001), progression-free (*p* < 0.001), and overall survival (*p* < 0.001) than GINI < 1562. For each survival endpoint, the association between GINI and survival outcomes appeared independent of other confounding variables (*p* < 0.05 for each). The novel GINI index effectively stratified patients with stage IIIC NSCLSC into two distinct subgroups, demonstrating significant differences in both median and long-term survival rates.

**Abstract:**

Background: We sought to determine the prognostic value of the newly developed Global Immune-Nutrition-Inflammation Index (GINI) in patients with stage IIIC non-small cell lung cancer (NSCLC) who underwent definitive concurrent chemoradiotherapy (CCRT). Methods: This study was conducted on a cohort of 802 newly diagnosed stage IIIC NSCLC patients who underwent CCRT. The novel GINI created first here was defined as follows: GINI = [C-reactive protein × Platelets × Monocytes × Neutrophils] ÷ [Albumin × Lymphocytes]. The receiver operating characteristic (ROC) curve analysis was used to determine the optimal pre-CCRT GINI cut-off value that substantially interacts with the locoregional progression-free (LRPFS), progression-free (PFS), and overall survival (OS). Results: The optimal pre-CCRT GINI cutoff was 1562 (AUC: 76.1%; sensitivity: 72.4%; specificity: 68.2%; Youden index: 0.406). Patients presenting with a GINI ≥ 1562 had substantially shorter median LRPFS (13.3 vs. 18.4 months; *p* < 0.001), PFS (10.2 vs. 14.3 months; *p* < 0.001), and OS (19.1 vs. 37.8 months; *p* < 0.001) durations than those with a GINI < 1562. Results of the multivariate analysis revealed that the pre-CCRT GINI ≥ 1562 (vs. <1562), T4 tumor (vs. T3), and receiving only 1 cycle of concurrent chemotherapy (vs. 2–3 cycles) were the factors independently associated with poorer LRPS (*p* < 0.05 for each), PFS (*p* < 0.05 for each), and OS (*p* < 0.05 for each). Conclusion: The newly developed GINI index efficiently divided the stage IIIC NSCLSC patients into two subgroups with substantially different median and long-term survival outcomes.

## 1. Introduction

The most recent version of the tumor-node-metastasis (TNM) staging system of the American Joint Committee on Cancer (AJCC 8th edition) estimates that one-third of all non-small cell lung cancers (NSCLC) are diagnosed at a locally advanced, unresectable disease stage. Adjuvant durvalumab immunotherapy increased 3-year overall survival (OS) rates to 57% in a subset of unresectable stage III NSCLC patients who responded to standard concurrent chemoradiotherapy (CCRT) [1,2]. However, many countries continue to restrict the use of adjuvant immunotherapy due to its high cost, and 5-year OS rates in CCRT-only trials have remained less than 18% [3].

The TNM staging system is considered the most reliable and resilient predictor of outcome for patients with stage III unresectable NSCLC who undergo CCRT treatment. However, despite using similar CCRT protocols, there are significant differences in tumor control and survival outcomes among these patients, likely due to the staging framework’s sole focus on the size and local growth of the primary tumor and its regional metastasis status [4]. Regrettably, the TNM framework ignores essential host- or tumor-related characteristics such as performance status, the number of positive lymph nodes and stations, gross tumor volume, genetic alterations, and biological variables [3,5,6,7]. However, these variables can influence the results dramatically, either positively or negatively [3]. Hence, it is of utmost importance to identify and incorporate additional biological indicators as supplementary tools to the TNM staging system, which will enhance the prognostic classification of patients and potentially facilitate the implementation of more personalized treatment approaches.

A growing body of research suggests that chronic inflammation, the seventh hallmark of cancer, plays a crucial role in virtually all steps of carcinogenesis and disease progression, from uncontrolled cellular proliferation to overt metastasis [8,9]. Typically, the inflammatory response involves neutrophils, monocytes, platelets, and lymphocytes, along with the cytokines/chemokines secreted by these cells and the acute-phase reactant proteins manufactured by various cells in the body, such as albumin and C-reactive protein (CRP). Numerous researchers have therefore examined the prognostic value of these blood-borne cells and proteins, either as single parameters or in various combinations, in patients with locally advanced NSCLC [10,11,12,13,14,15]. The findings of these studies and meta-analyses consistently indicated that these indices had substantial prognostic value in these patient groups.

Despite the availability of ample and favorable preclinical and clinical evidence, no previous studies have combined neutrophils, monocytes, platelets, lymphocytes, albumin, and CRP in a single comprehensive predictive or prognostic index. In this context, we hypothesized that the novel Global Immune-Nutrition-Inflammation Index (GINI), which integrates these cellular and biochemical inflammation indicators, could improve the prognosis prediction for patients with stage IIIC NSCLC. Therefore, the primary objective of this retrospective cohort investigation was to evaluate the predictive significance of the newly created GINI index in patients with stage IIIC NSCLC who underwent definitive CCRT at our medical facility.

## 2. Materials and Methods

### 2.1. Ethics, Consent, and Permissions

The study design employed in this research was retrospective and received approval from the institutional review board at Baskent University Medical Faculty. The study adhered to the principles and standards outlined in the Declaration of Helsinki and the Guidelines for Good Clinical Practice and their subsequent amendments. Before CCRT, all patients needed to provide their signed informed consent for the analysis of their clinical and blood test data in research and the publication of any pertinent findings.

### 2.2. Patient Population

We identified patients with stage IIIC (AJCC 8th ed.) NSCLC who received CCRT between January 2010 and December 2020 with 60 Gy thoracic RT and at least one concurrently administered chemotherapy cycle through a retrospective search of institutional records. Patients were required to meet the following criteria to be eligible for this study: aged between 18 and 80 years, have an Eastern Cooperative Oncology Group (ECOG) performance score of 0–1, body mass index (BMI > 18.5 kg/m^2^), pathological proof of NSCLC [adenocarcinoma (AC) or squamous cell carcinoma (SCC)], stage IIIC disease according to computerized tomography (CT) and 18F-fluorodeoxyglucose positron emission tomography-CT (PET-CT) findings, available pre-CCRT brain magnetic resonance imaging (MRI) scans, and complete blood count and biochemistry test results. Exclusion criteria for this study included the existence of malignant pleural/pericardial effusion, involvement of contralateral supraclavicular lymph nodes, a prior history of RT or chemotherapy, as well as inadequate pulmonary, cardiac, renal, or hepatic functions. Similarly, to minimize the potential impact of immune or inflammatory diseases and the medications used to treat them on the variables under investigation, individuals who had chronically active immune or inflammatory disorders, confirmed active infections, recent administration of steroids or antibiotics within the preceding 30 days, or blood transfusions within the past ninety days were excluded from this study.

### 2.3. Treatment Details 

Under the institution’s care standard for this patient population, no patients received elective nodal RT. We defined all target volumes, total and fractional RT dosages, normal tissue tolerance dose limits, and concurrent chemotherapy regimens using the same principles previously reported [16,17]. Each patient’s thoracic RT plans were carried out using co-registered diagnostic CT and PET-CT data. Each patient received thoracic RT using the intensity-modulated RT (IMRT) technique. The treatment plan involved delivering a total dose of 60 Gy over 30 fractions (2 Gy/fraction, 5 days per week). In addition, all participants received 1 to 3 cycles of cisplatin/carboplatin concurrently with either docetaxel, paclitaxel, or vinorelbine.

### 2.4. Calculation of the Global Immune-Nutrition-Inflammation Index (GINI)

The novel GINI, first introduced here, was formulated as follows:

$$\mathrm{GINI}=\frac{\mathrm{CRP}\;\times \;M\;\times \;P\;\times \;N}{\mathrm{Albumin}\;\times \;L}$$

where CRP, M, P, N, and N represent the C-reactive protein, monocytes, platelets, neutrophils, and lymphocyte counts measured before the first fraction of the prescribed CCRT dose.

### 2.5. Patient Follow-Up and Response Assessments

Although the study had a retrospective design, all assessments were prospectively documented. After completing the course of the CCRT, patients were subjected to regular follow-up appointments. These visits were scheduled at intervals of 3 months during the initial two-year period and subsequently adjusted to occur every 6 months or as deemed necessary. The therapeutic response was assessed following the 1999 guidelines established by the European Organization for Research and Treatment of Cancer (EORTC). This assessment involved serial complete blood count and biochemistry tests, as well as PET-CT or chest CT scans (in cases where there was compelling evidence of a complete metabolic response on PET-CT) [18]. Only the cases presenting with a clinical suspicion of distant metastasis or local disease recurrence underwent supplemental radiologic and nuclear medicine imaging examinations.

### 2.6. Statistical Analysis

In this retrospective study, the primary objective was to investigate if the novel GINI index, which was first introduced here, could effectively classify stage IIIC NSCLC patients into two groups with statistically significant differences in OS (the time between the start of CCRT and the date of death or the last visit). The secondary objectives were locoregional progression-free survival (LRPFS: the time between the first day of treatment and recurrence or progression at the primary tumor site and/or ipsilateral and/or contralateral hilum/mediastinum) and progression-free survival (PFS: the time between the first day of CCRT and the date of the first observation of disease progression of any type, death, or the last visit). The continuous quantitative variables were described by calculating their medians and ranges, while the categorical variables were described by analyzing their frequency distributions. We used the Chi-square test, Student’s *t*-test, Pearson’s exact test, or Spearman’s correlation estimates to compare the frequency distributions of the tested groups. Receiver operating characteristic (ROC) curve analysis was employed to ascertain the presence of an optimal GINI cutoff that effectively separates the study population into two distinct groups, exhibiting significantly disparate OS, LRPFS, and PFS outcomes. Log-rank tests were utilized to compare the OS, LRPFS, and PFS Kaplan-Meier curves. Only variables that were significant in the univariate analysis were included in the multivariate analysis. The Cox proportional hazards model was used to determine the independent significance of these variables. All comparisons were conducted using a two-tailed test, and any *p*-value < 0.05 was deemed statistically significant.

## 3. Results

The retrospective database search of our institution’s medical records revealed 802 patients with stage IIIC NSCLC who received definitive CCRT between January 2010 and December 2020 and met the inclusion criteria. The baseline patient and disease characteristics for the entire research population are displayed in Table 1. Overall, the CCRT protocol was well tolerated, as evidenced by rates of grade 3–4 acute toxicity of 34.4% [29.9% (N = 240) grade 3 and 4.5% (N = 36) grade 4] and grade 5 acute toxicity of 0% (Table 2). Late grades 3–4 and 5 toxic events were evident in 63 (7.9%) and 7 (0.9%) patients, respectively. Late toxic deaths were attributed to intractable broncho-esophageal fistula (N = 3), fatal pulmonary hemoptysis (N = 3), and radiation-induced pneumonia (N = 1).

With a median follow-up period of 27.2 months [95% confidence interval (CI): 16.8–37.6 months], 208 patients (25.9%) were still alive, and 126 (15.7%) were disease-free at the time of the final analysis. The median and 5-year LRPFS, PFS, and OS estimates for the entire population were 14.9 months (95% CI: 14.2–15.6 months) and 12.4%, 11.2 months (95% CI: 10.6–11.8 months) and 10.4%, and 23.7 months (95% CI: 22.1–25.3 months) and 17.9%, respectively (Table 2). Locoregional failure (LRF) and distant metastases (DM) were observed in 430 (53.6%) and 673 (83.9%) patients, respectively (Table 2).

**Figure 1:**
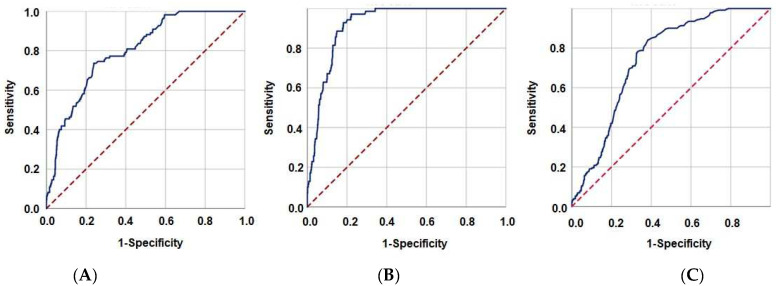
Receiver operating characteristic curve analysis results depicting the association between the pretreatment Global Immune-Nutrition-Inflammation Index values and survival outcomes: (**A**) Locoregional progression-free survival, (**B**) Progression-free survival, and (**C**) Overall survival.

The search for optimal GINI cutoffs that could potentially affect treatment outcomes showed significant results for LRPFS at 1556 [area under the curve (AUC): 80.1%; sensitivity: 78.4%; specificity: 75.2%; Youden index: 0.536], for PFS at 1569 (AUC: 91.9%; sensitivity: 91.6%; specificity: 81.8%; Youden index: 0.734), and for OS endpoints at 1562 (AUC: 76.1%; sensitivity: 72.4%; specificity: 68.2%; Youden index: 0.406), as shown in Figure 1. Due to the proximity of the three cutoffs, we decided to use the value of 1562 as the common cutoff for all endpoints to divide patients into two groups for comparative analyses: Group 1: GINI < 1562 (N = 364) and Group 2: GINI ≥ 1562 (N = 438). As expected, while the monocyte, platelet, neutrophil, and CRP counts were significantly higher in the GINI ≥ 1562 group, the albumin and lymphocyte levels were considerably higher in its GINI < 1562 counterpart. Despite the comparable distribution of other baseline patient and disease features, as well as treatment characteristics, between the two cohorts, patients in the GINI ≥ 1562 cohort exhibited significantly higher rates of acute (42.2% vs. 25.0%; *p* = 0.009) and late (10.3% vs. 4.9%; *p* = 0.037) grades 3–4 CCRT-related toxicities in comparison to their GINI < 1562 counterparts (Table 2).

**Figure 2:**
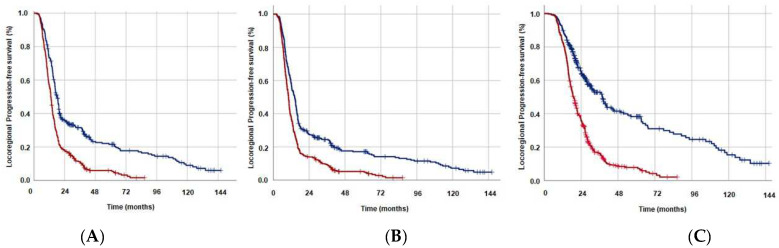
Survival outcomes per Global Immune-Nutrition-Inflammation Index (GINI) groups (Dark blue: GINI < 1562; Red: GINI ≥ 1562): (**A**) Locoregional progression-free survival, (**B**) Progression-free survival, and (**C**) Overall survival.

The analysis of disease control outcomes indicated that the rates of LRF (60% vs. 45.9%; *p* = 0.003) and DM (89.9% vs. 76.6%; *p* = 0.001) were substantially higher in the GINI ≥ 1562 group in comparison to the GINI < 1562 group (Table 2). Similarly, patients in the GINI ≥ 1562 group had significantly shorter median times to LRF (15.2 vs. 21.1 months; *p* < 0.001) and DM (12.1 vs. 16.9 months; *p* = 0.002) (Table 2). Intergroup survival comparisons between the two GINI cohorts using Kaplan-Meier plots and log-rank tests demonstrated that the median LRPFS (13.3 vs. 18.4 months; *p* < 0.001), PFS (10.2 vs. 14.3 months; *p* < 0.001), and OS (19.1 vs. 37.8 months; *p* < 0.001) were all significantly shorter in the GINI ≥ 1562 cohort (Table 2 and Figure 2). As depicted in Table 2, the matching 5-year LRPFS, PFS, and OS rates were also numerically lower in the GINI ≥ 1562 group. 

Apart from the high pre-CCRT GINI values (≥1562 vs. <1562), univariate analyses encompassing all pretreatment and treatment features showed that higher T-stage (4 vs. 3; *p* < 0.05 for each endpoint) and a lower number of concurrent chemotherapy cycles (1 vs. 2–3; *p* < 0.05 for each endpoint) were the other factors linked to significantly inferior LRPFS, PFS, and OS outcomes (Table 3). The multivariate Cox regression analyses conducted on these three variables demonstrated that each variable retained its independent and statistically significant impact on the outcomes of LRPFS, PFS, and OS (Table 3).

## 4. Discussion

The current study was designed to investigate the prognostic robustness of the newly created GINI index, which integrates factors related to immunity, nutrition, and inflammation, in stage IIIC NSCLC patients receiving definitive CCRT. The findings of our study provide novel evidence that the GINI index can categorize these patients into two distinct groups, showing significant differences in terms of LRPFS (*p* < 0.001), PFS (*p* < 0.001), and OS (*p* < 0.001) outcomes. These findings underscore the underappreciated prognostic significance of immune, nutritional, and inflammatory markers, which may aid in more accurate stratification of stage IIIC patients when used as a supplement to the TNM staging framework.

The novel and most significant contribution of our study to the stage IIIC NSCL literature was identifying pre-CCRT GINI values as statistically potent predictors of patients’ prognoses. Accordingly, we discovered that patients with a pre-CCRT GINI ≥ 1562 had significantly inferior median LRPFS (*p* < 0.001), PFS (*p* < 0.001), and OS (*p* < 0.001) than their comparators with GINI < 1562. Moreover, the 5-year LRF (60% vs. 45.9%; *p* = 0.003) and DM (89.9% vs. 76.6%; *p* = 0.001) estimates were significantly worse in the GINI ≥ 1562 group. These results suggest that the novel and nearly all-in-one immune, inflammatory, and nutritional index, GINI, may be useful in the prognostic stratification of stage IIIC NSCL patients into two essentially distinct prognostic groups following standard radical CCRT. Because the LRPFS, PFS, and OS rates in the GINI ≥ 1562 patients are nearly identical to those reported for stage IV NSCLC patients receiving palliative chemotherapy with/without RT [19], these findings may aid in determining appropriate treatments in an individualized manner if GINI is used in conjunction with the TNM staging framework. The current lower rates of locoregional and distant disease control infer that inflammation-induced chemo- and radio-resistance of the index primary and occult systemic metastases, which were undetectable by currently available advanced staging methods such as CT, MRI, and PET-CT scans, may be the cause of the GINI ≥ 1562 cohort’s poor outcomes [4,20]. Given the limitations of the TNM staging system and the conventional staging tools in distinguishing between stage IV patients and stage IIIC patients with a GINI ≥ 1562, it would be prudent to consider the incorporation of more advanced staging techniques, such as liquid biopsy procedures, into the standard staging protocols of such patients. 

The present study also revealed a noteworthy discovery: a clear connection between high pretreatment GINI values and higher rates of acute (42.2% vs. 25.0%; *p* = 0.009) and chronic (10.3% vs. 4.9%; *p* = 0.037) grade 3–4 CCRT-related toxicities. While GINI is a new index tested for this purpose, our research findings accord well with the limited biomarker reports in patients with NSCLC and small-cell lung cancer (SCLC) [21,22]. The study conducted by Go et al. presented evidence indicating that SCLC patients with an Onodera’s prognostic nutritional index (OPNI) of <40 had a reduced tolerance to chemotherapy and an unfavorable prognosis [21]. The incidence of treatment-related toxicity leading to the premature termination of initial therapy was more prevalent in the lower OPNI groups (high: OPNI > 45, intermediate: OPNI 40–45, low: OPNI < 40), with rates of 5.8%, 21.3%, and 25.6%, respectively (*p* < 0.001). In a separate study, Gioulbasanis and colleagues evaluated the Glasgow Prognostic Score (GPS) to ascertain its predictive value for toxicity and response in 50 NSCLC and 46 SCLC patients who received platinum-based chemotherapy [22]. In this study, mucositis (*p* = 0.004), neurotoxicity (*p* = 0.038), neutropenia (*p* = 0.02), dose reductions (*p* = 0.005), the need for granulocyte colony-stimulating factor support (*p* = 0.005), toxicity-related treatment discontinuation (*p* = 0.001), and chemotherapy-related toxic deaths (*p* = 0.013) were reported to be associated with GPS. While the OPNI and GPS studies in lung cancer patients complement our findings, our study is unique as it includes a larger cohort of only stage IIIC NSCLC patients treated with a standard protocol, namely definitive CCRT.

The novel GINI provides a practically all-in-one immunological, inflammatory, and nutritional biomarker by incorporating cellular and biochemical components of these processes, which individually and cumulatively influence therapy response, patient prognosis, and treatment tolerance in cancer patients [20]. The pathophysiological mechanisms behind the significant relationships between a high pre-CCRT GINI and reduced survival and increased toxicity outcomes in patients with LA-NSCLC remain unclear. Still, it is possible to construct theoretical perspectives by examining the specific immune, inflammatory, and nutritional functions of the cellular and biochemical components in the GINI formula. GINI can be partitioned in various ways; however, it may be more logical to investigate its biochemical and cellular components separately. Hence, the novel GINI can be reformulated as the product of CAR (CRP-to-albumin ratio) and PIV (pan-immune-inflammation value). The novel GINI can also be expressed as CAR × SIRI × P or CAR × SII × M, where SIRI, P, SII, and M stand for the systemic immune response index, platelets, systemic immune-inflammation index, and monocytes, respectively. The CAR, the biochemical component of the novel GINI, is a valuable metric for evaluating the status of systemic inflammation and nutrition, which influence the treatment tolerance and prognosis of patients with solid cancers, including NSCLC [23,24,25]. Deng et al. conducted a meta-analysis to investigate the relationship between CAR and overall OS in patients with lung cancer [24]. The analysis included four studies with a total of 1257 patients. The pooled analysis of all patients (HR: 1.63; *p* < 0.001), patients undergoing surgery (HR: 2.64; *p* < 0.001), and patients receiving chemotherapy (HR: 1.75; *p* = 0.004) demonstrated that elevated CAR values were associated with poor OS outcomes. Jia-min et al. confirmed these results in a subsequent trial that included 130 patients with lung adenocarcinoma [25]. The PIV, first proposed by Fucà et al., refers to the cellular component of the GINI, which comprises all four major blood-borne immune and inflammatory cell types in its formulation: platelets, monocytes, neutrophils, and lymphocytes [26]. There is compelling evidence suggesting a strong link between pretreatment levels of PIV and the subsequent outcomes in colorectal, breast, esophageal, pancreatic, oral cavity, small-cell and non-small-cell lung cancers, Merkel cell carcinomas, glioblastoma multiforme, and malignant melanoma patients [27,28,29,30,31,32,33]. Previously, Zeng et al. [30] and Kucuk et al. [31] demonstrated that high pretreatment levels of PIV were linked with substantially worse survival outcomes in SCLC patients treated with a combination of chemotherapy and immunotherapy or definitive CCRT. Concerning NSCLC patients, only one study has investigated the prognostic worthiness of PIV in this patient population [34], which explored the prognostic significance of PIV in 94 patients with advanced anaplastic lymphoma kinase (ALK)-positive NSCLC who had received first-line ALK inhibitors. Among all blood-borne biomarkers examined, only the elevated pretreatment PIV exhibited a significant association with PFS (HR = 2.90; *p* < 0.001) and OS (HR = 4.70; *p* < 0.001) in the multivariate analysis. Comparable outcomes were also observed in LA-NSCLC patients who underwent definitive concurrent chemoradiotherapy (CCRT) when utilizing SIRI and SII, two variations derivable from the GINI [4,20,35,36]. Although additional research is necessary to confirm the prognostic robustness of the novel GINI introduced in this study, indirect evidence suggests that GINI is a nearly all-in-one biological marker that combines easily accessible blood-borne biochemical and cellular constituents with immune, inflammatory, and nutritional functions that determine the treatment tolerance and prognosis of LA-NSCLC patients after CCRT.

From a pathophysiological perspective, the GINI can provide insights into the patient’s nutritional status and susceptibility to developing cancer cachexia over the course of CCRT treatment or during the subsequent follow-up period. The potential of GINI is linked to the incorporation of two nutritional indices, namely albumin and CRP, which are also recognized as indicators of cancer cachexia. During exacerbated inflammatory states, there is an inverse quantitative relationship between albumin and CRP. In response to inflammation, CRP levels rise quickly due to increased hepatic anabolism. Conversely, increased levels of CRP and its end products, tumor necrosis factor-alpha (TNF-α) and interleukin-6 (IL-6), lead to decreased levels of the malnutrition marker albumin. Increased CRP and decreased albumin levels are independently associated with a worse prognosis in advanced NSCLC [37,38]. Increased individual levels of CRP and lower levels of albumin, indicating a high CRP-to-albumin ratio (a component of the GINI formula), have also been recognized among the biochemical parameters utilized for a conclusive cancer cachexia definition in the Washington consensus reported by Evans et al. [39]. Hence, although all patients in our study had a baseline BMI of at least 18.5 kg/m^2^, GINI ≥ 1562 patients were probably experiencing a precachectic health state, which may have later turned into irreversible cancer cachexia with significant detrimental effects on survival outcomes [40]. However, additional research should investigate the accessibility of an indisputable correlation between GINI levels and cancer cachexia before definitive conclusions can be drawn on this topic of critical importance.

The strengths of the present study encompass several aspects. First, it benefited from a large sample size of patients diagnosed with stage IIIC NSCLC. Second, it assured uniformity by employing PET-CT staging consistently across all participants. Third, the study adopted a standard strategy known as CCRT for treatment and provided supportive and nutritional care tailored to the specific needs of patients throughout the treatment process. Fourth, the study employed a standardized methodology for assessing treatment response across all patients. Fifth, the study measures the components of the GINI at a consistent time point relative to the initiation of CCRT. Of course, our study did have some limitations. First, our results may have been influenced by unintentional biases frequently observed in single-institutional retrospective studies. Second, we did not examine any potential correlations between the GINI groups and other biomarkers, such as the cytokines and chemokines produced and released by the specific GINI components. Hence, we might have wasted an opportunity to demarcate the plausible mechanisms underlying the association between the GINI measures and survival outcomes. Third, we derived our results from a single snapshot of pre-CCRT GINI measurements, disregarding that all six components of novel GINI are dynamic biomarkers that can fluctuate widely during and after CCRT. Future research should therefore focus on GINI dynamics to establish a more influential cutoff that may be useful for the more accurate prognostic stratification of such patient groups. Fourth, it is possible that the lack of an internal validation group may have hindered the ability to accurately determine the prognostic significance of the new GINI index. Therefore, it may be beneficial in future research to include validation cohorts to clarify this issue in these patient populations; otherwise, further research should externally validate our results. Fifth, unintended variations between the salvage therapies may have altered the results reported here in favor of one GINI group, necessitating additional research to reach more conclusive remarks regarding this specific topic. Accordingly, it is critical to view the findings presented here as hypothetical until substantial proof from further research becomes available.

## 5. Conclusions

The present study’s findings indicate that the newly developed GINI index successfully classified the patient cohort into two subgroups, each exhibiting significantly different median and long-term survival outcomes. If confirmed by additional research, the novel GINI index may aid in the more precise classification of stage IIIC patients, thereby facilitating the selection of the most appropriate oncologic treatment for them in the era of individualized oncologic therapies. 

## Figures and Tables

**Table 1:** Pretreatment patient and disease characteristics at presentation.

Covariate	All Patients (N = 802)	GINI < 1562 (N = 364)	GINI ≥ 1562 (N = 438)	*p*-Value
Median age, y (range)	66 (28–79)	68 (28–79)	65 (34–79)	0.63
Age group, y (%)				0.74
≤70 years	565 (70.4)	261 (71.7)	304 (69.4)	
>70 years	237 (29.6)	103 (28.3)	134 (30.6)	
Gender, n (%)				0.82
Female	265 (33.0)	123 (33.8)	142 (32.4)	
Male	537 (67.0)	241 (66.2)	296 (67.6)	
ECOG, n (%)				0.46
0	223 (27.8	107 (29.7)	116 (26.5)	
1	579 (72.2)	257 (70.6)	322 (73.5)	
Median body surface area, m^2^, (range)	1.76 (1.51–2.16)	1.71 (1.53–2.16)	1.79 (1.51–2.08)	0.51
Median BMI, kg/m^2^, (range)	21.6 (18.9–38.7)	22.5 (19.4–38.4)	21.1 (18.9–37.2)	0.33
BMI category, n (%)				0.67
Normal weight (18.5–24.9 kg/m^2^)	496 (61.8)	219 (60.1)	277 (63.2)	
Overweight (25.0–29.9 kg/m^2^)	214 (26.7)	102 (28.0)	112 (25.6)	
Obese (≥30 kg/m^2^)	92 (11.5)	43 (11.9)	49 (11.2)	
Median albumin, g/L, (range)	36.2 (21.3–56.8)	41.4 (27.3–56.8)	30.1 (21.3–48.2)	0.003
Median CRP, mg/L, (range)	3.2 (0.2–36.8)	1.9 (0.2–23.8)	5.8 (0.7–36.8)	<0.001
Median monocyte count, 10^3^	0.74 (0.14–2.62)	0.32 (0.21–1.43)	1.11 (0.14–2.62)	<0.001
Median platelet count, 10^3^	257 (123–478)	223 (169–362)	297 (123–478)	0.006
Median neutrophil count, 10^3^	7.62 (2.22–18.94)	5.93 (2.34–18.94)	9.87 (2.22–18.72)	0.002
Median lymphocyte count, 10^3^	2.33 (0.79–5.98)	4.37 (2.1–5.98)	1.42 (0.79–5.98)	<0.001
Smoking history, n (%)				0.91
Absent	39 (4.9)	17 (4.7)	22 (5.0)	
Present	763 (95.1)	347 (95.3)	416 (95.0)	
Histology, n (%)				0.59
SCC	327 (40.8)	151 (41.5)	176 (40.2)	
AC	475 (59.2)	213 (58.5)	262 (59.8)	
T-stage, n (%)				0.33
T3	457 (57.0)	211 (58.0)	246 (52.7)	
T4	345 (43.0)	153 (42.0)	192 (47.3)	

Abbreviations: GINI: Global immune-nutrition-inflammation index; ECOG: Eastern Cooperative Oncology Group; BMI: Body mass index; CRP: C-reactive protein-SCC: Squamous cell carcinoma; AC: Adenocarcinoma; T-stage: Tumor stage.

**Table 2:** Treatment characteristics, toxicity outcomes, and survival results for the entire research cohort and per pan-immune-inflammation value group.

Outcome	All Patients (N = 802)	GINI < 1562 (N = 364)	GINI ≥ 1562 (N = 438)	*p*-Value
Concurrent Ctx cycles, n (%)				0.23
1	183 (22.8)	67 (18.4)	116 (26.5)	
2–3	619 (77.3)	297 (80.6)	322 (73.5)	
Maintenance Ctx, n (%)				0.029
Absent	574 (71.6)	241 (66.2)	333 (76.0)	
Present	228 (28.4)	123 (33.8)	105 (24.0)	
Acute Grade 3–4 toxicity, n (%)				0.009
Absent	526 (65.6)	273 (75.0)	253 (57.8)	
Present	276 (34.4)	91 (25.0)	185 (42.2)	
Late Grade 3–4 toxicity, n (%)				0.037
Absent	739 (92.1)	346 (95.1)	393 (89.7)	
Present	63 (7.9)	18 (4.9)	45 (10.3)	
Grade 5 toxicity, n (%)				0.88
Absent	795 (99.1)	361 (99.2)	434 (99.1)	
Present	7 (0.9)	3 (0.8)	4 (0.9)	
LRF, n (%)				0.003
Absent	372 (46.4)	197 (54.1)	175 (40.0)	
Present	430 (53.6)	167 (45.9)	263 (60.0)	
Median time to LRF, mo (95% CI)	17.2 (13.4–21.0)	21.1 (17.4- 24.8)	15.2 (12.6–17.8)	<0.001
DM, n (%)				<0.001
Absent	129 (16.1)	85 (23.4)	44 (9.9)	
Present	673 (83.9)	279 (76.6)	394 (89.9)	
Median time to DM, n (%)	13.4 (10.8–16.0)	16.9 (14.6–19.2)	12.1 (9.7–14.5)	0.002
LRPFS				<0.001
Median, mo.	14.9	18.4	13.3	
5-year (%)	12.4	21.1	6.0	
PFS				<0.001
Median, mo.	11.2	14.3	10.2	
5-year (%)	10.4	17.1	5.2	
OS				<0.001
Median, mo.	23.7	37.8	19.1	
5-year (%)	17.9	32.1	7.9	

Abbreviations: PIV: Pan-immune-inflammation value; IMRT: Intensity-modulated radiotherapy; Ctx: Chemotherapy; DM: Distant metastasis; PFS: Progression-free survival; OS: Overall survival; mo.: months.

**Table 3:** Results of univariate and multivariate analysis.

Characteristic	Patients (N)	Median LRPFS (Months)	Univariate *p*-Value	Mulivariate *p*-Value	Median PFS (Months)	Univariate *p*-Value	Mulivariate *p*-Value	Median OS (Months)	Univariate *p*-Value	Mulivariate *p*-Value
Age group			0.63	-		0.42	-		0.57	-
≤70 years	565	15.2			12.2			24.5		
>70 years	237	14.1			10.7			23.1		
Gender			0.56	-		0.37	-		0.32	-
Female	265	15.4			11.8			24.6		
Male	537	14.2			10.7			22.9		
Median body surface area, m^2^			0.37	-		0.62	-		0.32	-
<1.76	376	14.7			11.1			22.6		
≥1.76	426	15.7			11.9			24.8		
Median BMI, kg/m^2^			0.19	-		0.26	-		0.17	-
<21.6	367	14.6			10.7			22.3		
≥21.6	435	15.9			12.1			25.5		
BMI category, n (%)			0.42	-		0.49	-		0.28	-
Normal weight	496	14.4			10.8			22.4		
Overweight	214	15.8			11.3			24.7		
Obese	92	15.2			11.8			23.6		
ECOG			0.22	-		0.19	-		0.17	-
0	223	16.3			12.8			25.9		
1	579	13.9			10.9			23.0		
Smoking history			0.71	-		0.86	-		0.58	-
Absent	39	15.6			11.5			24.3		
Present	763	14.7			11.1			23.5		
Histology			0.37	-		0.58	-		0.28	-
SCC	327	13.8			10.7			22.4		
AC	475	15.6			11.6			24.0		
T-stage			0.008	0.011		0.006	0.009		<0.001	0.004
T3	457	17.2			13.3			27.9		
T4	345	13.5			10.4			22.0		
Concurrent Ctx cycles			0.005	0.009		0.007	0.008		0.003	0.005
1	183	12.9			10.6			22.8		
2–3	619	17.0			14.2			26.2		
Maintenance Ctx			0.79	-		0.61	-		0.53	-
Absent	574	14.7			10.7			22.7		
Present	228	15.3			11.8			24.6		
GINI group			<0.001	<0.001		<0.001	<0.001		<0.001	<0.001
<1562	364	19.4			14.3			37.8		
≥1562	438	13.3			10.2			19.1		

Abbreviations: PFS: Progression-free survival; OS: Overall survival; ECOG: Eastern Cooperative Oncology Group; SCC: Squamous cell carcinoma; AC: Adenocarcinoma; T-stage: Tumor stage; N-stage: Nodal stage; IMRT: Intensity-modulated radiotherapy; Ctx: Chemotherapy; PIV: Pan-immune-inflammation value.

## Data Availability

The Present data belongs to and is stored at the Baskent University Faculty of Medicine it cannot be shared without permission. For researchers who meet the following criteria for access to confidential data, contact the Baskent University Corporate Data Access/Ethics Board: adanabaskent@baskent.edu.tr.
